# Assessment of bacterial diversity during composting of agricultural byproducts

**DOI:** 10.1186/1471-2180-13-99

**Published:** 2013-05-07

**Authors:** Piyush Chandna, Lata Nain, Surender Singh, Ramesh Chander Kuhad

**Affiliations:** 1 Lignocellulose Biotechnology Laboratory, Department of Microbiology, University of Delhi South Campus, Benito Juarez Road, New Delhi, 110 021, India; 2 Division of Microbiology, Indian Agricultural Research Institute, New Delhi, 110 012, India

**Keywords:** Agricultural by-products, Compost, Bacterial diversity, Culturable, Phylogeny

## Abstract

**Background:**

Composting is microbial decomposition of biodegradable materials and it is governed by physicochemical, physiological and microbiological factors. The importance of microbial communities (bacteria, actinomycetes and fungi) during composting is well established. However, the microbial diversity during composting may vary with the variety of composting materials and nutrient supplements. Therefore, it is necessary to study the diversity of microorganisms during composting of different agricultural byproducts like wheat bran, rice bran, rice husk, along with grass clippings and bulking agents. Here it has been attempted to assess the diversity of culturable bacteria during composting of agricultural byproducts.

**Results:**

The culturable bacterial diversity was assessed during the process by isolating the most prominent bacteria. Bacterial population was found to be maximum during the mesophilic phase, but decreased during the thermophilic phase and declined further in the cooling and maturation phase of composting. The bacterial population ranged from 10^5^ to 10^9^ cfu g^-1^ compost. The predominant bacteria were characterized biochemically, followed by 16S rRNA gene sequencing. The isolated strains, both Gram-positive and Gram-negative groups belonged to the order *Burkholderiales*, *Enterobacteriales, Actinobacteria*les and *Bacillales*, which includes genera e.g. *Staphylococcus*, *Serratia*, *Klebsiella*, *Enterobacter*, *Terribacillus, Lysinibacillus Kocuria*, *Microbacterium*, *Acidovorax* and *Comamonas*. Genera like *Kocuria*, *Microbacterium*, *Acidovorax*, *Comamonas* and some new species of *Bacillus* were also identified for the first time from the compost made from agricultural byproducts.

**Conclusion:**

The use of appropriate nitrogen amendments and bulking agents in composting resulted in good quality compost. The culture based strategy enabled us to isolate some novel bacterial isolates like *Kocuria*, *Microbacterium*, *Acidovorax* and *Comamonas* first time from agro-byproducts compost. These bacteria can be used as potential compost inoculants for accelerating composting process.

## Background

Lignocellulosic agricultural byproducts are well known for their use as soil conditioners in the form of compost. According to conservative estimates, around 600–700 million tones (mt) of agricultural waste including 272 mt of crop residues [1]; 40–50 mt of municipal solid waste (MSW) and 500–550 mt of animal dung [2] are available in India every year for bioconversion to compost.

Composting is an intense microbial process leading to decomposition of the most biodegradable materials for further humification [3,4]. Successful composting depends on a number of factors that have both direct and indirect influence on the activities of the microorganisms. Tiquia *et al*. [5] included the type of raw material being composted, its nutrient composition and physical characteristics such as bulk density, pH, and moisture content etc. as the important factors. Moreover, Fracchia *et al*. [6] also observed that various other factors influenced the microbial colonization of finished products, i.e., (i) origin and composition of the initial substrates, (ii) previous process conditions and (iii) substrate quality of the finished product.

For the composting processes, the importance of microbial communities is well established [7]. Studies on bacterial population, actinobacteria and fungi during composting have been reported extensively [8]. Liu *et al.*[9] reported that there were several molecular approaches, which provide powerful adjuncts to the culture-dependent techniques. A known powerful tool, namely PCR has been used for bacterial identification and its classification at species level [10]. PCR targeting the 16S rRNA gene sequencing is used extensively to study the prokaryote diversity and allows identification of prokaryotes as well as the prediction of phylogenetic relationships [11]. The analyses of rRNA genes encoding for the small subunit ribosomal RNA (for bacteria, 16S rRNA) [12-14] have recently dramatically increased our knowledge about the contribution of different bacteria to various compost production phases.

Molecular approach to characterize and classify microbial communities by cultivation methods has switched to the genetic level, and the analysis of community structure has become possible only with further need to address the cultivation approach for a systematic analysis. Cultivation based techniques have allowed merely a glimpse of microbial diversity because only an estimated 1% of the naturally occurring bacteria has been isolated and characterized so far [15]. Even though, recent advances in culture independent molecular approaches based on rRNA or genomic approaches have improved the knowledge of microbial ecosystems, the isolation of bacterial species in pure culture remains to be the only way to fully characterize them, both for their physiological and catabolic properties. Moreover, the unculturable bacteria identified using recent molecular techniques cannot be used as compost inoculant for improving composting process. Therefore, culture-dependent methods are still a powerful tool. These viable fractions (grown to a detectable level on agar based medium) form only a small part of the total microorganisms, but they can still be used for comparison of data representing different times of the year or different areas [16]. So, it is imperative to study in-depth the culturable bacterial diversity so as to identify some new bacteria which can be applied for better and quick compost preparation. Besides composting, bacteria isolated from compost have been used by many researchers for others applications as well [17,18].

In the traditional methods of composting some pathogenic bacteria survived, this was probably because of an inadequate aeration and lack of building-up of relatively high temperature. Moreover, the prevailing conditions might have prevented some of the indigenous microorganisms to colonize and degrade plant wastes. As a result, the final composts obtained from such an unimproved method are generally poor in quality. It has therefore become highly exigent to develop an alternative technique for producing good quality compost using locally available lignocellulosic biomass and bulking agents. This paper describes an attempt to identify specific microorganisms involved in the degradation of plant materials with the aim of studying the succession of bacterial population during composting in order to exploit the isolated bacteria in future for diverse uses such as compost inoculants, enzyme production, biocontrol agents.

## Results

### Physicochemical characteristics of compost

**Figure 1:**
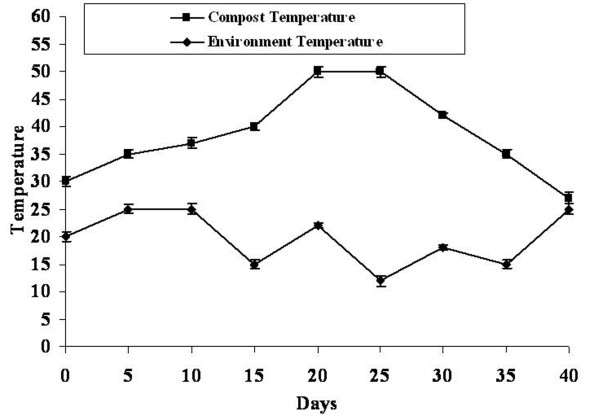
Temperature in the compost heap and environment during composting period.

The pile and environmental temperatures were monitored during the entire period of composting (Figure 1). Initial temperature of the heap after mixing was 30°C. Within a week, the pile temperature reached to 37°C. However, the temperature increased to 40°C after 15 days and remained the same for four days, thereafter, which it rose to 50°C on 20^th^ day and remained static for next few days. However, as composting proceeded, the temperature of the pile dropped to 45°C by the 30^th^ day and fell further, but stabilized at 27°C (near to ambient) by the sixth week. After that, the pile was left uncovered for cooling for the next ten days.

During the present study, the substrates mixtures showed an initial electrical conductivity (EC) of 3.8 dS m^-1^. It reached upto 4.9 dS m^-1^ with progressive degradation upto 40 days. The pH of the compost heap remained 7.5 during first 30 days of the process, and thereafter it declined to 7.0 and continued till 50^th^ day.

### Chemical characteristics

The changes in organic carbon (C), total nitrogen (N), the C: N ratio, phosphorus and potassium varied considerably during composting (Table 1). The organic C decreased, whereas total nitrogen, phosphorus and potassium increased with time. Finally C: N ratio was observed to be stabilized at 11:1 at the end of composting during 40–50 days.

**Table 1:** Physicochemical properties of the agricultural byproducts compost

	**Physical properties**		**Chemical properties**											
			**Metals concentration**											
Days	Moisture	C (%)	N (%)	C: N	P (%)	K (%)	Ca (g kg^-1^ dw)	Mg (g kg^-1^ dw)	S (g kg^-1^ dw)	Na (g kg^-1^ dw)	Zn (mg kg^-1^ dw)	Cu (mg kg^-1^ dw)	Mn (mg kg^-1^ dw)	Fe (mg kg^-1^ dw)
0	50.5	17.3	1.3	31.1	0.8	1.0	13.0	8.4	2.3	1.3	86.6	33.0	266.9	93.0
10	50.4	16.0	1.4	26.6	0.9	1.0	13.2	8.9	2.3	1.8	90.4	34.2	268.4	100.6
20	50.3	14.1	1.4	21.0	1.0	1.1	13.5	9.2	2.5	2.1	98.2	39.5	270.6	112.3
30	50.3	13.0	1.4	15.5	1.1	1.1	13.9	9.8	2.5	2.4	101.3	44.3	281.0	129.9
40	50.1	11.4	1.5	11.7	1.2	1.1	13.9	10.2	2.5	2.5	124.6	50.7	286.0	134.8
50	50.1	11.4	1.5	11.4	1.2	1.1	13.9	10.2	2.5	2.5	124.6	50.7	286.2	134.8
(%)	negligible	-50.9	+9.6		+33.1	+15.0	+5.9	+17.6	+8.0	+48.0	+30.5	+34.9	+6.9	+31.0

Here ‘-’indicates decrease in concentration and ‘+’ indicates increase in the concentration; counts upto 40 days, and next 10 days remained for stabilization.

### Total micronutrients

There was a significant increase in nutrients e.g. Na, Cu, Zn, Mg, S, Mn, Fe and Ca during composting. The respective average values of various metal contents varied considerably from the beginning to end of composting (Table 1).

### Changes in viable bacterial population during composting

The number of mesophilic bacteria increased rapidly in first ten days, the count of mesophilic bacterial count was 1.7- 2.84 × 10^9^cfu g^-1^. However, the thermophilic bacteria were dominant from 11–32 days of composting, with count in between 10^8^ to10^7^cfu g^-1^. Finally, mesophilic population stabilized between 10^6^ to 10^5^ cfu g^-1^ during the cooling and maturation phase (33–40 days).

### Morphological, biochemical and molecular characterization of isolates

**Figure 2:**
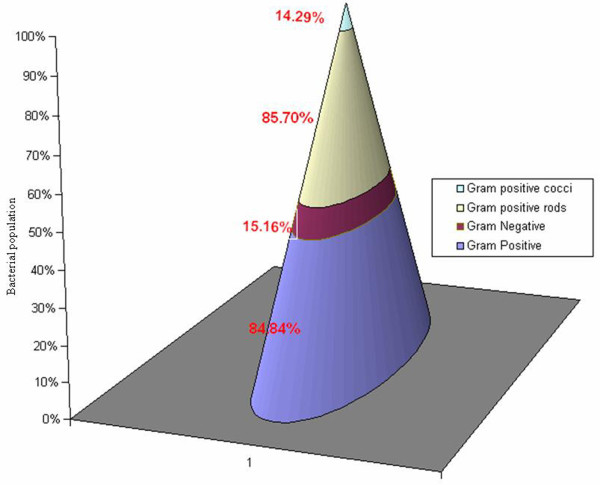
Characteristics feature of the isolated strains.

The most predominant bacterial isolates were picked up and morphologically different colonies were selected for further studies (Table 2). A total of thirty-three bacteria were subsequently purified and subjected to morphological, biochemical and molecular characterization. Interestingly, 84.8% isolates were Gram-positive, out of which 85.7% were rods and 14.3% cocci, whereas, the remaining 15.2% of the isolates were Gram-negative and all them were rods (Figure 2). The bacterial cultures were tentatively identified on the basis of Bergey’s Manual of Systematic Bacteriology (Table 3).

**Table 2:** Morphological feature of the bacteria isolated from the compost

**Medium**	**Nutrient agar**														
Laboratory designation/ No.	red	17	H	J, G, BC	32	26	M	QR	D, 30, Q, 21	actin 1, Y, 3	actin 5, actin 2, actin 6	X, IN,8, 38, N	B, A, Q	L, 14, PQ	31, 31 (a)
Shape	circular	round	circular	circular	round	round	round	circular	circular	irregular	circular	circular	irregular	oval	oval
Margin	entire	entire	entire	entire	wavy	spreading	uniformly edged	entire	regular	entire	entire	entire	regular	regular	regular
Elevation	slightly convex	convex	convex	slightly convex	convex	convex	slightly convex	slightly convex	convex	convex	convex	convex	undulate	convex	convex
Texture	smooth	wet	smooth	smooth	smooth	smooth	smooth	smooth	smooth	smooth	smooth	smooth	smooth	smooth	dull
Color with special characters (Pigmentation)	red	grey colored colonies	slightly gummy colonies	white or creamish white	creamish white	slighly brown	white or creamish white	shiny	white	white	white	white	white	creamish white	white
Opacity	translucent	translucent	opaque	opaque	translucent	opaque	translucent	opaque	translucent	opaque	opaque	opaque	translucent	opaque	translucent
Gram’s Staining	(-ve) with 0.5-0.8 μm in length; with rounded ends	(-ve) with 1.0 mm dia; coccoid rods	(-ve) with 0.3 -1.0 μm; straight rods arranged in short chains	(+ve) with 1–2 mm in dia; large cocci	(-ve) with 0.3-1.5 μm in length; slightly curved rods	(-ve) with 0.2-0.7 x 1.0-5.0 μm; rods straight to slightly curved	(+ve) with 1.0–1.5 *m*m in dia; coccoid occurring in pairs and in tetrads	(+ve) with 1 mm in dia; small slunder rods	(+ve) with 0.7 – 2.5 μm in size; rods	(+ve) with 1–2 mm in dia; rods with short chains ended	(+ve) with 1 mm in dia; rods with short ends	(+ve) rod with short in chains with 1–2 mm in diameter	(+ve) 0.5 -1.0 μm; straight rods arranged in short chains	(+ve) with 1–2 μm; rods with short rounded ended	(+ve) with 1 to 2 μm; short rods

**Table 3:** Biochemical profiling of a bacteria: + = positive, - = negative, ± = variable, R= rods, C= cocci

**Laboratory no./ Designation**	**J**	**8**	**30**	**G**	**PQ**	**A**	**38**	**14**	**31 (a)**	**BC**	**red**	**H**	**17**	**actin 6**	**3**	**QR**	**B**	**M**	**D**	**14**	**26**	**X**	**32**	**Y**	**21**	**N**	**IN**	**Q**	**actin 5**	**actin 2**	**actin 1**	**‘31’ and ‘l’ data not shown**
Morphology	+ C	+ R	+ R	+ C	+ R	+ R	+ R	+ R	+ R	+ C	- R	- R	-R	+ R	+ R	+ R	+ R	+ C	+ R	+ R	- R	+ R	- R	+ R	+ R	+ R	+ R	+ R	+ R	+ R	+ R	
Motility	-	-	+	-	+	+	+	+	+	-	-	-	-	+	-	-	-	+	-	+	+	-	-	-	+	+	-	+	-	-	-	
Methyl red	-	-	-	-	-	-	-	-	±	-	-	-	-	-	±	-	-	-	-	±	+	-	-	-			+		-	-	-	
Voges-Proskauer	-	-	-	-	-	-	-	-	-	-	-	-	-	-	-	-	-	-	-	-	-	-	-	-	-	-	-	-	-	-	-	
Citrate	+	-	±	+	-	+	-	-	+	-	+	+	+	-	-	+	+	+	+	-	±	+	+	-	±	-	+	-	±	+	±	
Indole	-	-	-	-	-	-	-	-	-	-	+	-	-	-	±	-	-	-	-	-	-	-	-	-	-	-	-	-	-	-	-	
Morphology	+ C	+ R	+ R	+ C	+ R	+ R	+ R	+ R	+ R	+ C	- R	- R	-R	+ R	+ R	+ R	+ R	+ C	+ R	+ R	- R	+ R	- R	+ R	+ R	+ R	+ R	+ R	+ R	+ R	+ R	
Motility	-	-	+	-	+	+	+	+	+	-	-	-	-	+	-	-	-	+	-	+	+	-	-	-	+	+	-	+	-	-	-	
Methyl red	-	-	-	-	-	-	-	-	±	-	-	-	-	-	±	-	-	-	-	±	+	-	-	-			+		-	-	-	
Voges-Proskauer	-	-	-	-	-	-	-	-	-	-	-	-	-	-	-	-	-	-	-	-	-	-	-	-	-	-	-	-	-	-	-	
Citrate	+	-	±	+	-	+	-	-	+	-	+	+	+	-	-	+	+	+	+	-	±	+	+	-	±	-	+	-	±	+	±	
Indole	-	-	-	-	-	-	-	-	-	-	+	-	-	-	±	-	-	-	-	-	-	-	-	-	-	-	-	-	-	-	-	
Glucornidase	-	-	-	-	-	-	-	-	-	+	-	-	-	-	-	-	-	-	-	+	±	-	-	-	-	+	-	-	-	-	-	
Nitrate reduction	+	-	+	-	+	+	+	+	+	+	+	+	+	-	+	+	-	+	+	-	+	+	-	+	+	+	+	+	+	+	+	
Lysine decarboxylase	+	+	-	-	-	-	-	-	-	+	+	+	+	-	-	+	+	+	+	-	+	+	+	±	-	-	+		-	+	+	
Lactose	-	-	-	-	+	-	-	-	-	-	-	+	-	-	+	-	-	±	-	-	+	±	-	±	-	+	-	-	-	-	-	
Glucose	+	-	+	+	+	+	-	-	-	+	+	+	+	-	+	+	-	+	-	-	+	-	+	+	+	+	+	+	+	+	+	
Sucrose	+	+	+	+	+	+	-	-	-	+	+	+	+	-	+	+	-	+	+	-	+	+	+	+	-	-	+	+	+	+	+	
Sorbitol	+	-	+	+	+	+	-	-	-	+	±	+	-	-	+	-	-	+	-	+	+	±	-	+	-	+	+	-	-	-	-	
Ornithine decarboxylase	-	-	-	-	-	-	-	-	-	-	+	+	+	-	-	+	+	+	-	-	+	+	+	-	-	-	+	-	-	+	+	
Urease	+	-	-	+	-	-	-	-	-	+	-	-	-	-	-	-	-	-	-	-	-	-	-	-	-	-	-	-	-	-	-	
Deamination	-	-	-	-	-	-	-	-	-	-	-	-	-	-	-	-	-	-	-	-	-	-	-	-	-	-	-	-	-	-	-	
H_2_S production	-	-	-	-	-	-	-	-	-	-	-	-	-	-	-	-	+	-	-	-	-	-	-	-	-	-	-	-	-	-	-	
ONPG	-	-	-	-	-	-	-	-	-	-	+	+	+	-	-	-	-	-	+	-	+	-	+	-	-	-	-	-	-	+	-	
Xylose	-	-	-	-	-	-	-	-	-	-	-	-	-	-	-	-	-	-	-	-	-	-	-	-	-	-	-	-	-	-	-	
Maltose	+	-	-	+	+	-	-	+	+	+	-	+	-	-	+	+	-	+	-	-	+	+	+	+	+	-	-	-	-	+	+	
Fructose	+	-	+	+	+	+	-	-	-	+	-	+	-	-	+	+	-	+	+	-	+	+	+	+	-	-	+	+	+	+	+	
Dextrose	+	-	+	+	+	+	-	-	-	+	+	+	-	-	+	+	-	+	-	-	+	-	+	+	+	+	+	+	+	+	+	
Galactose	-	+	+	+	+	+	+	+	+	+	+	+	-	+	+	+	-	+	-	+	+	+	+	+	+	+	-	+	-	-	-	
Raffinose	-	+	-	-	+	+	+	+	±	+	-	+	-	+	+	-	-	+	-	+	+	-	+	-	+	+	-	+	-	-	-	
Trehalose	+	+	+	+	-	-	-	-	-	+	-	+	-	+	+	+	-	+	-	-	+	+	+	+	-	-	+	-	+	-	+	
Mellibiose	-	+	-	-	-	-	-	-	-	+	-	+	-	+	+	-	-	+	-	+	+	+	+	-	+	-	-	-	-	-	-	
L-Arabinose	+	-	+	+	-	-	-	-	+	-	-	+	+	+	+	+	-	-	+	+	+	-	-	+	-	-	-	-	+	+	-	
D-Arabinose	-	-	-	-	-	-	-	-	-	-	-	+	-	-	+	-	-	-	-	-	+	+	-	-	+	+	+	-	+	-	+	
Inulin	-	-	-	-	-	-	-	-	-	-	-	-	-	-	+	-	-	-	-	-	-	-	-	-	-	-	-	-	-	-	-	
Sodium gluconate	-	-	-	-	-	+	+	+	-	-	-	-	-	+	+	-	-	-	-	-	+	-	-	+	+	-	-	-	-	-	-	
Glycerol	+	-	-	-	-	-	-	-	-	-	-	+	+	-	+	+	-	-	+	-	+	+	+	+	-	-	+	-	-	-	-	
Salicin	+	-	+	-	+	-	+	-	+	-	-	-	-	+	+	-	-	-	+	+	-	-	-	+	+	-	+	-	-	-	-	
Glucosamine	-	-	+	+	+	+	+	+	-	-	+	-	-	+	+	+	-	-	-	+	-	-	-	+	+	+	-	±	-	-	-	
Dulcitol	-	-	-	-	+	-	+	-	-	-	-	-	-	+	-	-	-	+	-	-	-	-	-	-	+	-	-	+	-	-	-	
Inositol	-	-	+	-	+	-	-	+	-	+	-	+	-	+	-	+	-	+	-	-	-	+	-	+	+	+	-	-	-	-	-	
Mannitol	+	-	-	+	+	-	-	+	-	+	+	+	-	-	+	+	-	-	+	-	+	+	+	+	-	+	-	+	-	-	-	
Adonitol	-	-	-	-	-	-	-	-	-	-	±	+	-	-	-	-	-	-	-	-	-	±	-	-	-	-	-	-	-	-	-	
α-methyl-D-glucoside	-	-	-	-	-	-	+	+	-	+	-	-	-	-	+	-	-	-	-	+	-	-	+	-	+	+	-	-	-	-	-	
Ribose	+	-	+	+	+	+	+	+	+	+	+	+	-	+	-	-	-	-	-	+	+	+	+	+	-	-	+	-	-	-	-	
Rhamnose	-	-	-	-	-	-	+	-	-	-	-	+	-	+	+	+	-	-	-	+	-	-	-	+	-	+	+	+	-	-	-	
Cellobiose	-	-	-	-	-	-	-	-	-	-	-	-	-	-	±	-	-	-	-	-	-	-	-	±	-	-	-	-	-	-	-	
Melezitose	+	-	+	-	+	-	+	-	+	+	-	-	-	+	+	-	-	-	-	+	-	-	-	+	+	+	-	+	-	-	-	
α-methyl- D-mannoside	-	-	-	-	-	-	-	-	-	-	-	-	-	+	-	-	-	-	-	+	-	-	-	-	-	+	-	+	-	-	-	
Xylitol	-	-	-	-	-	-	-	-	-	-	-	-	-	-	-	-	-	-	-	-	-	-	-	-	-	-	-	-	-	-	-	
Aesculin hydrolysis	-	+	+	-	+	-	-	-	-	-	+	+	+	+	-	+	+	-	+	-	+	-	-	-	+	-	+	-	+	+	-	
Malonate	+	-	+	+	+	+	-	-	-	+	-	+	-	+	-	-	+	+	-	+	-	-	-	-	+	-	-	-	-	-	-	
Sorbose	-	-	-	-	-	-	-	+	-	+	-	-	-	+	-	-	-	-	-	+	-	-	-	-	-	-	-	-	-	-	-	
Mannose	-	-	+	-	-	-	-	-	-	-	+	+	-	+	+	+	-	+	-	-	+	+	-	+	+	-	+	-	+	+	-	
Propable organism	*Staphylococcus* sp.	*Bacillus* sp.	*Bacillus* sp.	*Staphylococcus* sp.	*Bacillus* sp.	*Bacillus* sp.	*Bacillus* sp.	*Bacillus* sp.	*Bacillus* sp.	*Staphylococcus* sp.	*Serratia* sp.	*Klebsiella* sp.	*Enterobacter* sp*.*	*Bacillus* sp.	*Bacillus* sp.	*Microbacterium* sp.	*Bacillus* sp.	*Kocuria* sp.	*Terribacillus* sp.	*Bacillus* sp.	*Acidovorax* sp.	*Bacillus* sp.	*Comamonas* sp.	*Bacillus* sp.	*Bacillus* sp.	*Bacillus* sp.	*Bacillus* sp.	*Bacillus* sp.	*Bacillus* sp.	*Bacillus* sp.	*Bacillus* sp.	*Bacillus* sp.

### Identification of culturable bacteria isolated from compost

Marked changes in the profiling patterns of bacteria between the initial, mid and final stages of the composting process were observed. The changes in the structure of bacterial community were analyzed on the basis of 16S rRNA gene sequence chronometer from day one to end of composting. The amplified PCR products of bacterial 16S rRNA genes were sequenced partially.

**Figure 3:**
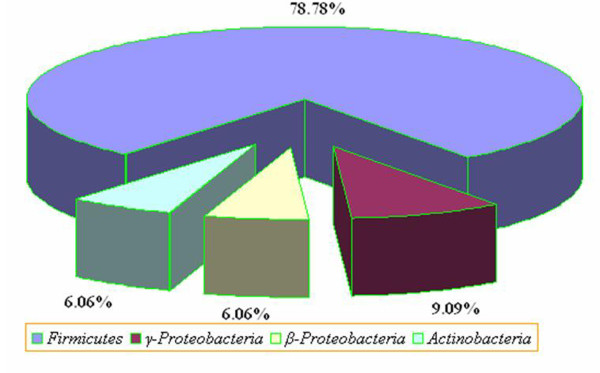
Distribution of the bacterial strains isolated from compost identified by 16S rDNA chronometer.

All sequences were compared with 16S rRNA gene sequences present in the Genebank using BLAST and their percentage similarity was also compared and recorded in Table 4. The majority of the bacterial isolates (78.8%) were affiliated with Firmicutes (especially the genera *Bacillus* sp., *Terribacillus* sp. and *Lysinibacillus* sp. etc.), whereas only 9.1, 6.1 and 6.1% of bacterial isolates were affiliated to the members of γ-proteobacteria, β-proteobacteria and actinobacteria, respectively (Figure 3). Apart from spore forming *Bacilli* other genera in the compost were *Staphylococcus*, *Serratia*, *Klebsiella*, *Enterobacter*, *Microbactrium*, *Kocuria*, *Acidovorax* and *Comamonas*.

**Table 4:** Characterization of the dominant bacteria through molecular signature of 16S rRNA genes amplified from the genomic DNA extracted from the bacterial isolates isolated from the composting during different phase

**Laboratory designation**	**Morphological features (Gram staining) & Phylogenetic group**	**Isolate name with Accession no**	**16S r DNA similarity (nucleotide identity)**	**Accession no. of nearest neighbor**	**Temperature & phase**
J	+,cocci; firmicutes	*Staphylococcus sciuri* Durck1 AM778178	94%	EF204304.1	30°C & Mesophilic
8	+,rods ; firmicutes	*Bacillus pumilus* Durck14 AM778191	95%	AY647298.1	
30	+,rods ; firmicutes	*Bacillus subtilis* Durck10 AM778185	91%	AY879290.1	
G	+,cocci; firmicutes	*Staphylococcus sciuri* Durck9 AM778188	98%	AB188210.1	
PQ	+,rods ; firmicutes	*Bacillus subtilis* Durck7 AM778184	90%	AY881638.1	
A	+,rods; firmicutes	*Bacillus subtilis* Durck12 AM778189	99%	AY881638.1	
38	+,rods; firmicutes	*Bacillus pumilus* Durck8 AM778187	99%	AB244427.1	
14	+,rods; firmicutes	*Bacillus flexus* Durck6 AM778183	96%	EF157301.1	
31(a)	+,rods ; firmicutes	*Bacillus flexus* Durck15 AM778192	96%	DQ365587.1	
BC	+,cocci; firmicutes	*Staphylococcus sciuri* Durck16 AM884572	99%	AM778188.1	
red	-,rods; γ-proteobacteria	*Serratia marcescens* Durck24 FR865468	91%	EU781738.1	
H	-,rods ; γ-proteobacteria	*Klebsiella pneumoniae* Durck21 AM884577	96%	EU078621.1	
17	-,rods ; γ-proteobacteria	*Enterobacter sakazakii* Durck19 AM884575	97%	CP000783.1	35°C & Mesophilic
actin 6	+,rods ; firmicutes	*Bacillus pumilus* Durck23 AM884579	99%	DQ270752.1	
3	+,rods; firmicutes	*Bacillus cereus* Durck30 FR865474	94%	EU624445.1	
QR	+,rods; actinobacteria	*Microbacterium* sp. Durck18 AM884574	99%	AJ919993.1	
B	+,rods ; firmicutes	*Lysinibacillus fusiformis* Durck2 AM778179	91%	DQ333300.1	40°C & Thermophilic
M	+,cocci; actinobacteria	*Kocuria flavus* Durck22 AM884578	98%	EF675624.1	
D	+,rods; firmicutes	*Terribacillus halophilus* Durck28 FR865472	94%	AB243849.1	
14	+,rods; firmicutes	*Bacillus flexus* Durck5 AM778182	94%	DQ412062.1	
26	-,rods ; β-proteobacteria	*Acidovorax* sp. Durck31 FR865475	90%	AY258065.1	
X	+,rods; firmicutes	*Bacillus nealsonii* Durck26 FR865470	91%	DQ416782.1	
32	-,rods; β-proteobacteria	*Comamonas kerstersii* Durck29 FR865473	97%	AJ430348.1	45°C & Thermophilic
Y	+,rods; firmicutes	*Bacillus benzoevorans* Durck27 FR865471	96%	DQ416782.1	
21	+,rods; firmicutes	*Bacillus subtilis* Durck17 AM884573	98%	AY971362.1	
N	+,rods; firmicutes	*Bacillus pumilus* Durck13 AM778190	92%	AM778187.1	50°C & Thermophilic
IN	+,rods; firmicutes	*Bacillus pumilus* Durck3 AM778180	98%	AB301019.1	
Q	+,rods; firmicutes	*Bacillus subtilis* Durck11 AM778186	99%	AB301021.1	
actin 5	+,rods; firmicutes	*Bacillus subtilis* Durck4 AM778181	94%	AB244458.1	35°C & Cooling and Maturation
31	+,rods; firmicutes	*Bacillus composteris* RC1 Data not shown	Data not shown		
L	+,rods; firmicutes	*Bacillus southcampusis* RC2 Data not shown			
actin 2	+,rods; firmicutes	*Bacillus licheniformis* Durck20 AM884576	97%	DQ071561.1	
actin 1	+,rods; firmicutes	*Bacillus circulans* Durck25 FR865469	95%	AB189702.1	

**Figure 4:**
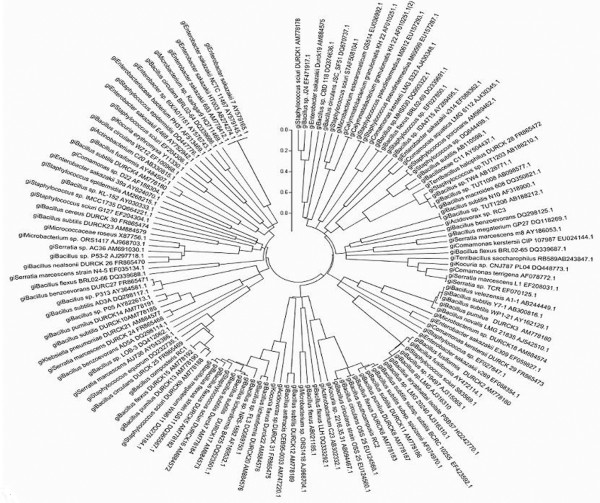
**Neighbour-joining unrooted tree depicting the phylogenetic relationship of the dominant bacteria among the related species of the genus.***Staphylococcus, Bacillus, Terribacillus, Lysinibacillus, Serratia, Klebsiella, Enterobacter, Microbacterium, Kocuria, Acidovorax* and *Comamonas* using MEGA 5 software.

Interestingly, genera like *Kocuria*, *Microbacterium*, *Acidovorax* and *Teribacillus* have been reported for the first time from the compost population from agricultural by-products. The heat generated during composting destroyed all pathogenic bacteria in the final mature compost and was found to be free from *Staphylococcus*, *Klebsiella*, *Enterobacter* and *Serratia*. The phylogenetic affiliation of compost isolates with their accession numbers and their nearest neighbors of the GenBank database are shown in (Figure 4 and Table 4).

## Discussion

Composting is a dynamic process affected by a large number of environmental and biological factors. Change in any of these factors greatly affects the quality of compost as well as the time required for composting. The present investigation demonstrated changes in temperature, physiochemical characteristics and bacterial population during composting process. This study also deals with the characterization of predominant bacterial genera isolated from different phases of composting.

Biddlestone and Gray [19] reported that the complexity of degraded plant materials and quality of the final product may depend upon the type of biomass. Therefore, various agricultural byproducts were used as raw material in order to provide an excellent substratum for the growth of microorganisms. All these supplements had high mineral and N content, which balance the relatively high C: N ratio of rice husk. Rice husk may supply K, Ca, Mg and other minerals along with C and silica [20]. In composting, C: N ratio was considered to be the most important parameter, as it reflects the extent of the bio-transformations that took place in the compost in chemical terms [21]. In the beginning of composting the C: N ratio of agricultural byproducts was 31.1 and it was decreased to 11.4 at the end of composting (Table 1). This decline might be because of reduction of C, which is obviously due to evolution of CO_2_ during degradation of organic matter and increase in N due to mineralization of organic-N compound. Brito *et al*. [22] also observed a decline in C: N ratio from 36 to 14 at the end of composting. The C: N ratio less than 12 during the solid phase was believed to be an indicator for the maturity of the compost [23,24].

The temperature regime in the compost indicated that the organic materials passed through different phases like mesophilic, thermophilic, cooling and maturation (Figure 1) as already reported by Ishii *et al*. [25]. The temperature started dropping in the compost pile once the material was stabilized, which also indicated that the pile was becoming anaerobic and should be aerated by turning [26]. Therefore, turning was performed first on 15^th^ day of composting, and then on every tenth day. The results indicated that processes like thorough mixing of the materials and turning enhanced the decomposition process. Moreover, if turning process failed to reheat the composting pile, it showed that the composting material was biologically stable [27].

### Nutrient status of mature compost

The results showed a significant increase in minerals (w w^-1^) in agricultural byproducts composting (Table 1) and no gradual fluctuations were observed after 40^th^ day. Janakiram and Sridevi [28] attempted the composting of Kattamanakku (*Jatropha curcas*) waste with slurries of cow dung by an aerobic composting method; the percentages of N, P, K, Na, Ca and Mg increased after 30 and 60 days of composting. The findings correlated with the present study. Similarly Felton *et al*. [29] reported that total P increased during the compost process. The metal concentrations like Cu, Mn and Zn increased rapidly during first 49 days of composting from swine (*Susdo mesticus*) feces [30]. The stability and solubility of various compounds in compost is influenced by the pH of the compost [31,32].

### Microbial population

Kell *et al*. [33] studied that at the simplest level, bacteria may be classified into two physiological groups: those that can, and those that cannot readily be grown to detectable levels *in vitro*. The viable count usually refers to the number of individual organisms in compost that can be grown to a detectable level, *in vitro* by forming colonies on an agar-based medium. However, the number of viable cells approximates to the number of colony forming units [34]. Changes in bacterial population were analyzed by cultivation-based method (cfu g^-1^) to reveal changes in the number of mesophilic and thermophilic bacteria during the composting process.

Hargerty *et al*. [35] reported that there was maximum increase in microbial population in the early stages of composting which was dependent on initial substrate used and environmental conditions of the composting. High content of degradable organic compound in the initial mixture might have stimulated microbial growth involved in self-heating during initial stage of composting [36]. An equivalent tendency does not occur with regard to mesophilic and thermophilic bacteria in the present study when the population density decreased from 10^9^ to 10^7^ cfu g^-1^. However from thermophilic to cooling and maturation phase, the gradual decrease in 10^7^ to 10^5^ cfu g^-1^ could be due to the unavailability of nutrients during maturation phase. During peak heating the bacterial populations declined by approximately 10-fold at 40°C and 100-fold at 50°C, followed by population growth at cooling phase, which decreased by 1000 fold as compared to the mesophilic (starting) phase of composting [7]. The Gram-positive bacteria dominated the composting process as they accounted for 84.8% of total population and the remaining 15.2% were Gram-negative as illustrated in Figure 2.

For bacteria, 16S rRNA gene sequence analysis is a widely accepted tool for molecular identification [37,38]. Franke-Whittle *et al*. [39] also investigated the microbial communities in compost by using a microarray consisting of oligonucleotide probes targeting variable regions of the 16S rRNA gene. During the present investigation, thirty three bacterial isolates were cultured, out of which twenty six isolates (78.8%) belonged to class firmicutes; two isolates (6.1 %) belonged to actinobacteria; three isolates (9.0 %) belonged to class γ-proteobacteria and the remaining two isolates (6.1%) showed sequence similarity to class β-proteobacteria (Figure 3). Table 4 and Figure 4 summarizes all the bacterial taxa reported in agricultural byproduct compost based on sequence similarity, which were categorized in four main classes: Firmicutes, β-proteobacteria, γ-proteobacteria and actinobacteria in concurrence with the findings of Ntougias *et al*. [40] and Chandna *et al*. [41].

The present study determined the microbial succession of the dominating taxa and functional groups of microorganisms, as well as the total bacterial activity during composting of agricultural byproducts, using incubation, isolation, and enumeration techniques. The bacterial population showed differences between mesophilic, thermophilic and maturing stages of compost. Ryckeboer *et al*. [7] analyzed the bacterial diversity and found that both Gram-positive and Gram-negative bacteria increased during the cooling and maturation phases of biowaste composting in compost bin. In the present study, the level of firmicutes increased markably during mesophilic phase, and then decreased during the next phase upto cooling and maturation. The number of actinobacteria count remained stable during mesophilic and thermophilic phase of composting. Population of β*-*proteobacteria remained insignificant in thermophilic phase whereas, the level of γ*-*proteobacteria increased slightly during mesophilic phase and then decreased markably during thermophilic phase. Similarly, Fracchia *et al*. [6] observed the prevalence of Gram-positive organisms belonging to the firmicutes and actinobacteria.

In the present study a few *Serratia*, *Enterobacter*, *Klebsiella* and *Staphylococcus* sp. were also isolated during early phase of composting. Silva *et al*. [42] also found *Serratia* sp. in bagasse and coast-cross straw during the first stage of composting. *Enterobacter* sp. was predominantly present at an early stage of composting process and died off at increased temperature [43] in accordance with the present study. Moreover, *Enterobacter* sp. is common in soil, water and even in compost too and mainly survives as saprophytes [44]. Strauch [45] found that the *Klebsiella* sp. was present at the beginning of thermophilic phase till the temperature was below 60°C. Similarly, Ahlawat and Vijay [46] also isolated *Staphylococcus* sp. from mushroom research farm compost at a wider temperature range (43–55°C). Importantly no pathogen could be detected during the curing phase of compost produced from agricultural byproducts. Thus our composting process also resulted in the eradication of pathogens, as has been reported by Danon *et al*. [47].

Heating is essential to enable the development of a thermophilic population of microorganisms, which is capable of degrading the more recalcitrant compounds, to kill pathogens and weed seeds [48]. *Bacillus* sp. was able to survive in the compost pile due to their property to form endospores during thermophillic stage. Various researchers investigated that *Bacillus* sp. was a predominant genera present throughout the composting process [25,49], and the most dominant bacterial taxon recovered from compost feedstock [50]. Additonally, *Kocuria* sp. was one of the isolates, cultured from present studied compost. Similarly, Vaz-Moreira *et al*. [51] also isolated *Kocuria palustris* from vermicompost from food wastes.

BLAST analysis (http://blast.ncbi.nlm.nih.gov/Blast.cgi) of 16S rRNA gene sequence revealed similarity to sequences of the species *Comamonas kerstersii*, a β-Proteobacterium of the Comamonadaceae family, as published in GenBank. Young *et al*. [52] isolated *Comamonas* sp. from food waste compost. It had the ability to metabolize complex organic compounds as energy sources for growth [53]. Moreover, *Comamonadaceae,* a new family encompassing the *Acidovorans*[54], was also recovered from agricultural byproduct compost. Pinel *et al*. [55] isolated *β*-proteobacterial *Acidovorax* sp. symbionts from the nephridia of four different species of earthworms. Pizl and Novokova [56] also showed the establishment of different kinds of relationship between earthworms and microbes. The nephridial symbionts form their own monophyletic group closely related to the genus *Acidovorax*[57]. The bacteria reduced the biodegradable organic content and help in mineralization of solid waste [58].

## Conclusion

The production of high quality compost can be enhanced by biological, physiochemical properties of raw material and compost inoculants. Present study indicated the usefulness of different nitrogen amendments and bulking agents for improved composting process to prepare high quality compost. These culture-based approaches taken in this study enabled us to isolate, for the first time, *Kocuria*, *Microbacterium*, *Acidovorax* and *Comamonas* from agricultural byproducts compost. However, in order to understand better the nature of bacterial communities associated with compost, the use of sequencing of 16S rRNA genes was used to describe the complete bacterial community composition. The new genera *Kocuria*, *Microbacterium*, *Acidovorax* and *Comamonas* identified from the compost can be used as compost inoculants for accelerating the composting process. Besides being prospected for degradation, they can be evaluated for their ability to produce hydrolytic enzymes and antimicrobial compounds etc.

## Methods

### Site selection, raw material for composting

The experiment was carried out at University of Delhi South Campus, New Delhi, India during the month of December 2006 and January 2007. The composting pile (1.50 × 0.90 × 0.80 m^3^) was prepared on a clean ground surface, covered with black polyethylene. The raw materials used for composting were rice bran (15 kg), wheat bran (10 kg), rice husk (10 kg) and other additives like grass and leaves (5 kg) each; ash (2.5 kg) was used as a bulking agent. Nitrogen (N) was enriched by amending with cow dung (25 kg), mustard oil cake (10 kg), cow urine (40 l) and molasses (4 l). To eliminate the pH variation, approximately 0.6% (w w^-1^) of calcium oxide was added to the compost raw materials during mixing. Table 5 depicts raw materials and their properties. The pile was turned manually on the 15^th^ day of composting and then after every 10^th^ day.

**Table 5:** Raw material and its properties

**Raw materials**	**C (%)**	**N (%)**	**C:N (ratio)**	**Hemicellulose (%)**	**Cellulose (%)**	**Lignin (%)**
Wheat bran	37.6	2.3	14:1	30.3	12.5	5.7
Rice husk	32.1	0.6	76:1	28.2	30.9	15.6
Rice bran	47.9	2.2	12:1	35.5	26.3	5.4
Molasses	26.1	1.0	27:1	48.3	33.4	19.2
Leaves	16.2	4.5	45:1	-	-	-
Grass clipping	30.3	3.6	15:1	28.6	24.5	-
Mustard oil cake	39.4	1.8	26:1	40.6	19.6	33.5
Cow dung	24.8	1.5	20:1	37.2	21.6	20.4
Cow urine	11.6	16.3	0.8:1	-	-	-

During the composting process, the temperature in the pile (5 to 30 cm from the top) was measured daily using a dry bulb thermometer. Similarly, the environment temperature was also recorded during composting near the pile. The samples were collected at every 10^th^ day for microbial and physicochemical analysis. The composting was terminated after 50 days. The duplicate samples were used to assess the consistency or reproducibility in the method.

### Physiochemical analysis of compost

Compost pH and electrical conductivity (EC) were measured by preparing a (1:5 w v^-1^ compost: water) mixture as described by Rhoades [59] and Blakemore *et al*. [60] respectively. The percent organic carbon (C) in the compost was determined by the wet digestion method outlined by Walkley and Black [61]. Total nitrogen (N) was estimated by Kjeldahl method [62] and total sulfur according to the method of Steinbergs [63]. The potassium was estimated by ammonium-acetate method [64]. The samples were analyzed for micronutrient by atomic absorption spectrophotometer (Model 3030, Perkin-Elmer, USA). Macronutrients like calcium (Ca), magnesium (Mg) were determined following the methodology of Moral *et al*. [65] and sodium (Na) by using the method of Thompson and Wood [66]. The trace metals; copper (Cu), zinc (Zn), iron (Fe) and manganese (Mn) were estimated by ICP-MS (Induced coupled plasma Mass Spectrometer) as per methodology of Koplık *et al*. [67]; Fingerová and Koplık [68]; Jenn-Hung and Shang-Lien [30], respectively.

### Isolation and enumeration of bacteria during composting

Bacteria were isolated from compost by serial dilution method by plating 100 μl of diluted suspension from each phase {the mesophile (30 and 35°C), thermophile (40 and 50°C), maturation and cooling phase (35 and 30°C) samples} were spread plated on nutrient agar (NA) plates. The plates were incubated at 30°C, 35°C, 40°C and 50°C for 24 h. Colonies were counted and populations were expressed in term of cfu g^-1^. Morphologically different colonies were purified on NA plates. All isolates and were preserved on slants at 4°C and glycerol stock at -20°C in 20% (v v^-1^). All chemicals and media were of molecular grade and procured from either Merck Pvt. Ltd or Himedia, India.

### Morphological, biochemical and molecular characterization

Presumptive identification was carried out by colony morphology and use of the first stage diagnostic biochemical tests for Gram-positive and Gram-negative bacteria. Further identification was carried out by standard biochemical tests by using Himedia tests kits (Hi motility™ and Assorted™ Biochemical kit, Hi Carbohydrate™ kit, Hi IMViC™ Biochemical test kit).

### Genomic DNA extraction, purification and quantification

Loopful of selected bacterial isolates were streaked and grown on NA plates at their relevant temperature and freshly grown isolates were used to inoculate in 50 ml of Luria-Bertani (LB) broth (Himedia, India). The broth cultures were grown at their respective temperature of the isolates with shaking at 200 rpm till the cultures reached OD_600_ of 0.4-0.5. Thereafter, cells were pelleted by centrifugation at 9167 × g for 10 min at 4°C and washed with TE buffer [10 mM Tris–HCl pH 8.0, 1 mM ethylenediaminetetraacetic acid (EDTA)] and pellets were either frozen (-20°C) for storage or used immediately for genomic DNA extraction by using the method of Sambrook & Russell [69].

DNA samples were quantified by running on agarose gel electrophoresis using 0.8% agarose gel in 1 × tris-boric acid EDTA (TBE) (89 mM tris pH 7.6, 89 mM boric acid, 2 mM EDTA) and visualized by ethidium bromide (0.5 μg ml^-1^) staining, to determine DNA size and to assess RNA contamination.

### PCR Amplification and sequencing

Amplifications were performed in 50 μl reaction mixture containing 75 ng of template DNA, 1-unit of i-Taq™ polymerase (NEB, UK), 2 mM MgCl_2_ (NEB, UK) , 2 μl of 10X PCR buffer, 0.1 mM dNTP (NEB, UK), 100 ng of each forward (8f’:5’-AGAGTTTGATCCTGGCTCAG-3’ [70]), and reverse (1542r’:5′-AAGGAGGTGATCCAGCCGCA-3’ [71]) primers. The amplification was carried out using G-strom thermal cycler (Labtech, UK). Amplification programme consisted of initial cycle of denaturation at 94°C for 5 min, 30 cycles of denaturation at 94°C for 1 min, annealing at 58°C for 1 min, initial extension at 72°C for 1 min 30 sec and final extension at 72°C for 7 min. Amplified products were electrophoresed at 5 Vcm^-1^ through 1.5% agarose gel containing 0.5 μg ml^-1^ ethidium bromide in 1xTBE electrophoresis buffer with 50 bp DNA Ladder (NEB, UK). The gels were visualized under UV illumination in Gel Documentation system 2000 (Biorad, Hercules CA, USA) and stored as TIFF file format. Sizes of the amplicons were estimated in comparison with 50bp DNA ladder (NEB, UK).

### Sequencing of 16S rRNA gene and phylogenetic analysis

The expected DNA band of 1.5 kb was excised from gel and purified using the gel elution kit (Sigma-Aldrich, USA) as per the manufacturer’s protocol. Sequencing reactions were carried out with a BigDye Terminator cycle sequencing kit (Applied Biosystems, USA), standard universal primer forward (8f’) and reverse (1542r’) primer and sequenced by using ABI Prism 3100 genetic analyzer (Applied Biosystems, USA). The sequences thus obtained were assembled and edited using Clone Manager Version 5 (http://www.scied.com/pr_cmbas.htm).

Database search was carried out for similar nucleotide sequences with the BLAST search of Non-reductant (NR) database (http://blast.ncbi.nlm.nih.gov/Blast.cgi) [72]). Partial length 16S rRNA gene sequences of strains closely related to the isolate were retrieved from NCBI for further analysis. For describing their phylogentic relationship, the partial 16S rRNA gene sequences were aligned by using Clustal_X [73]. A phylogenetic tree was constructed by means of neighbor-joining method using MEGA version 5 programme [74].

### Nucleotide sequences accession number

The nucleotide sequences of 16S rRNA were obtained and deposited in the GenBank database (EMBL, U.K.) and the accession numbers; AM778178-AM778192, AM884572-AM884579 and FR865468-FR865475 were assigned to their respective sequences.

## Abbreviations

(cfu g-1): Colony forming unit per gram; MSW: (Municipal Solid Wastes); PCR: (Polymerase Chain Reaction); EC: (Electrical Conductivity); NA: (Nutrient Agar); LB: (Luria-Bertani); OD: (optical density); (NEB): New England Biolab

## Competing interests

The authors declare that they have no competing interests.

## Authors’ contributions

RCK, PC, LN and SS planned the study. PC performed the experiments. PC and RCK analyzed the results. RCK, PC, LN and SS drafted the manuscript. All authors read and approved the final manuscript.
