# Correction: assessment of bacterial diversity during composting of agricultural byproducts

**DOI:** 10.1186/1471-2180-14-33

**Published:** 2014-02-11

**Authors:** Piyush Chandna, Lata Nain, Surender Singh, Ramesh Chander Kuhad

**Affiliations:** 1 Lignocellulose Biotechnology Laboratory, Department of Microbiology, University of Delhi South Campus, Benito Juarez Road, New Delhi 110 021, India; 2 Division of Microbiology, Indian Agricultural Research Institute, New Delhi 110 012, India

## Correction

After the publication of this work [1], we found that Table 4 is a duplication of Table 2 in our related publication [2] and some of the text within the methods section is also duplicated. We have now obtained permission to reuse this material with kind permission from Springer Science + Business Media: *Applied Microbiology and Biotechnology* (2013) **97** (15) p 6991–7003. Piyush Chandna, Sarita Mallik and Ramesh Chander Kuhad. Table 2. ©Springer-Verlag Berlin Heidelberg 2012.

We apologize for the inconvenience that this may have caused.
